# Peripheral refraction with different designs of progressive soft contact lenses in myopes

**DOI:** 10.12688/f1000research.9971.1

**Published:** 2016-11-22

**Authors:** Kareem Allinjawi, Sharanjeet-Kaur Sharanjeet-Kaur, Saadah Mohamed Akhir, Haliza Abdul Mutalib

**Affiliations:** 1 Optometry and Vision Science, Faculty of Health Science, University Kebangsaan Malaysia, Kuala Lumpur, Malaysia

**Keywords:** Progressive contact lens, myopia, hyperopic defocus, peripheral retina

## Abstract

**Aim:** The purpose of this study was to compare the changes in relative peripheral refractive error produced by two different designs of progressive soft contact lenses in myopic schoolchildren.

**Methods:** Twenty-seven myopic schoolchildren age between 13 to 15 years were included in this study. The measurements of central and peripheral refraction were made using a Grand-Seiko WR-5100K open-field autorefractometer without correction (baseline), and two different designs of progressive contact lenses (PCLs) (Multistage from SEED & Proclear from Cooper Vision) with an addition power of +1.50 D. Refractive power was measured at center and at eccentricities between 35º temporal to 35º nasal visual field (in 5º steps).

**Results:** Both PCLs showed a reduction in hyperopic defocus at periphery. However, this reduction was only significant for the Multistage PCL (p= 0.015), (Proclear PCL p= 0.830).

**Conclusion:** Multistage PCLs showed greater reduction in peripheral retinal hyperopic defocus among myopic schoolchildren in comparison to Proclear PCLs.

## Introduction

The most common type of eye refractive error is called myopia, which is considered a global health problem ^1^. With the beginning of 21 ^st^ century, Atchison *et al.* ^2^ 2006 and Mutti *et al.* ^3^ 2007 observed that myopic eyes have more hyperopic peripheral refraction than emmetropes in the horizontal visual field. Studies conducted by Smith and colleagues in monkeys have shown that not only the fovea, but also the peripheral retina, is capable of regulating the emmetropiszation process ^4– 6^. This indicates that the peripheral retina is important in determining ocular development and refractive error.

Studies have shown that conventional correction of myopia using spectacles lenses may increase hyperopic defocus in the periphery ^7, 8^. Hyperopic defocus worsens with a higher degree of myopia and eccentricity ^9^. In 2009, Tabernero *et al.* ^8^ suggested that by changing the peripheral optics of corrective devices, relative hyperopic defocus in myopic eyes could be inverted into peripheral relative myopia. This could be a possible strategy to counterbalance the unknown stimulus that triggers the eye elongation and subsequent progression of myopia.

Specially designed spectacle lenses ^10^ and contact lenses ^11^ have employed the change in peripheral optics of the optical design. Some commercially available progressive contact lenses (PCL) (dominant-design) intended for presbyopic patients might render a similar effect. The peripheral add power area, which was primarily intended to increase spherical aberration and depth of focus in presbyopic patients, has been shown to induce significant changes in the peripheral refractive error profile of the eye. Lopes-Ferreira *et al.* ^12^ found that a +3.00 D add dominant design Proclear progressive contact lens in 20 emmetropic and 28 myopic eyes inverted the hyperopic defocus to myopic defocus in the periphery. In another study by Rosén *et al.* ^13^, they were able to induce approximately 0.50 D of myopic defocus 30° using a +2.00 D lens in 1 myopic and three emmetropic patients. These studies were done in adults, and it is unclear if the hyperopic defocus could be inverted to myopic defocus in myopic children. This knowledge is important because myopia progression occurs mainly in children. The aim of this study was to compare the changes in relative peripheral refractive error produced by using two different designs of commercially available progressive soft contact lenses in myopic schoolchildren.

## Methods

Twenty-seven myopic schoolchildren (24 females, 3 males) aged between 13 and 15 years were recruited in this study. The purpose and procedure of the study were explained to all participants and their parents. Then, written informed consent was obtained before enrolment into the study. The study was conducted at the University Kebangsaan Malaysia (UKM) Optometry Clinic and Vision Science Lab. This research was approved by the Ethics Committee of the Universiti Kebangsaan Malaysia (UKM 1.5.3.5/244/NN-144-2013) and followed the tenets of the Declaration of Helsinki in using human subjects.

The inclusion criteria for this study were having visual acuity of 6/9 or better in both eyes, having normal ocular condition with a spherical component refractive error range between -3.00 and -6.00 D, astigmatism not more than -1.00 D and anisometropia of less than 1.50 D between both eyes. Children with manifest strabismus, amblyopia, any ocular conditions associated with myopia, a history of bifocal or progressive spectacle wear, orthokeratology contact lens wear, or those currently wearing soft contact lenses were excluded from participation in this study.

A comprehensive ocular examination, which included fundus evaluation, anterior segment assessment, and axial length calculation, was conducted by an experienced optometrist to select the candidates. The spherical equivalent refractive error ( *M*) for each subject was determined using non-cycloplegic objective and subjective refraction. An ultrasound A-scan (Tomey AL-2000) was used to measure axial length using a handheld probe. The final outcome was calculated as the mean of 5 measurements.

Central and peripheral refraction were measured using an open-view autorefractometer (Grand-Seiko WR-5100K,Grand Seiko Co., Ltd., Hiroshima, Japan). The examination room illumination was dimmed (mean of three measurements: 9.91 ± 1.73 lux, measured using a Topcon Luxmeter) in order to obtain a pupil size sufficiently large enough to measure peripheral retina without using dilatation drops. The measurement was obtained initially without contact lenses (WL), then re-measured again using Multistage progressive contact lenses (Multistage PCL, from SEED Co. Japan) and Proclear progressive contact lenses (Proclear PCL, from CooperVision) in random order. Subjects were masked to the type of each lens, while the practitioner was unmasked. The subjects were instructed to fixate on targets (green light laser) located at 4 metres arranged horizontally in the positions corresponding to eccentricities from 35° temporal to 35° nasal, in 5° steps. The straight ahead viewing technique was used in this study, in which the subjects rotated their eyes to view a series of fixation targets. Five refraction measurements were taken at each target fixation for the right eye only, while the left eye was occluded. For statistical analysis, the sphero-cylindrical refractive error measurements were converted into vector components of refraction *M*, *J*_0_, *J*_45_ using the equations recommended by Thibos *et al.* ^14^ *M*, *J*_0_ and *J*_45_ according to Fourier analysis,

*M* = sph + (cyl/2), *J*_0_ = (-cyl/2) cos (2 α), *J*_45_ = (-cyl/2) sin (2 α),

where sph, cyl and (α) represent sphere, cylinder and axis, respectively. Relative peripheral refractive error (RPRE) was calculated as the difference between eccentric peripheral refraction and central refraction. A one-way repeated measures ANOVA with Bonferroni’s post-hoc test was conducted to determine the changes in RPRE values for the mean spherical equivalent *M*, *J*_0_ and *J*_45_ components between the groups.

### Contact lenses design and materials

All subjects were fitted with Multistage PCL and Proclear PCL to their right eyes in random order on the same days. Lens powers fully corrected the central refractive error. The Multistage PCL used in the study was a biweekly soft contact lens made of 42% Group IV (ionic high water content) and 58% water content, with diameter of 14.2 mm and a base curve of 8.6 mm. The B-Design used in this study is spherical distance power at the centre zone (2.5 mm), a junction zone (2.5 mm to 3.5 mm) and a near zone (3.5 mm to 8.0 mm) with a maximum addition power of +1.50 D in the periphery.

The Proclear progressive D ^®^ design contact lens was a monthly disposable lens made from omafilcon A, with a water content of 62% and an overall diameter of 14.4 mm, with a base curve of 8.7 mm. The lens design has a 2.3-mm inner distance central spherical area, surrounded by an annular aspheric zone where the addition power increases gradually to reach its maximum power of +1.50D at 5 mm. There is a second spherical zone with a maximum addition power of +1.50D from 5 mm to 8 mm diameter. Table 1 illustrates the parameters of contact lenses used in this study.

**Table 1.** The designs and materials of Progressive contact lenses.

	Multistage PCL	Proclear PCL
Brand	2 weeks multistage	Proclear
Material	Group IV - Ioinc high water content	Omfilcon A
Modality	2 weeks	1 month
Power(s)	Type (B) Add +1.50	Type (D) Add +1.50
Base curve	8.6 mm	8.7 mm
Diameter	14.2 mm	14.4 mm
**a.)** Spherical distance zone diameter **b.)** Aspheric multifocal zone (width/Dia) **c.)** Spherical near zone (width/Dia)	2.5 ɸ mm 0.5mm/ 2.5 ɸ to 3.5 ɸ mm 2.25mm/ 3.5 ɸ to 8.0 ɸ mm	2.3 ɸ mm 1.35 mm/ 2.3 ɸ to 5.0 ɸ mm 1.75mm/ 5.0 ɸ to 8.5 ɸ mm
Water content	58%	62%

### Statistical analysis

Analysis was performed using SPSS statistical software version 20 (SPSS Inc., IL, USA). Only data from the right eye was analysed. A Shapiro–Wilk test was used to evaluate the normality of the data distribution. A paired t-test was used for paired comparisons of RPRE within each group at the different eccentricities with respect to the centre. When normality could not be assumed, the Wilcoxon signed-ranks test was used. The differences were considered statistically significant when the p value was lower than 0.05. Then, repeated measures analysis of variance (ANOVA) was performed to compare the RPRE between the different groups at the different eccentricities.

## Results

Raw data for ‘Peripheral refraction with different designs of progressive soft contact lenses in myopes’, 2016 Axial length measurement data is provided in the file “A-Scan”; Data for Table 2 “the mean spherical equivalent” and Table 3 “the relative peripheral refractive error” are provided as files “data Table 2” and “data Table 3”, respectively. Click here for additional data file. Copyright: © 2016 Allinjawi K et al. 2016 Data associated with the article are available under the terms of the Creative Commons Zero "No rights reserved" data waiver (CC0 1.0 Public domain dedication).

**Table 2.** Mean spherical equivalent ( *M* ± SD), horizontal astigmatism component ( *J*_0_ ± SD) and oblique astigmatism component ( *J*_45_ ± SD) for 27 eyes at different eccentricities under different conditions: without lens, Multistage PCL (Add +1.50D) and Proclear PCL (Add +1.50).

Eccentricity	*M*			*J*_0_			*J*_45_		
	WL	Multistage PCL	Proclear PCL	WL	Multistage PCL	Proclear PCL	WL	Multistage PCL	Proclear PCL
N35	-3.32 ±1.59	-0.41 ±1.25	0.02 ±1.35	0.11 ±0.84	-0.04 ±1.15	0.14 ±0.86	0.18±0.91	0.17±0.83	0.21±0.9
N30	-3.6 ±1.49	-0.73 ±1.01	-0.41 ±1.19	0.02 ±0.93	-0.08 ±0.78	0.15 ±0.6	-0.15±0.58	-0.1±0.7	0.21±0.83
N25	-4.09 ± 1.36	-1.13 ± 0.83	-0.78 ± 0.97	0.06 ±0.7	0.23 ±0.55	0.01 ±0.62	-0.18±0.58	-0.18±0.73	-0.05±0.59
N20	-4.31 ±1.09	-1.3 ±0.59	-1.14 ±0.76	0 ±0.36	0 ±0.42	-0.11 ±0.44	0.07±0.51	0.21±0.45	-0.1±0.41
N15	-4.41 ±1.11	-1.32 ±0.45	-1.16 ±0.62	0.01 ±0.28	-0.02 ±0.43	-0.01 ±0.3	0.05±0.37	-0.09±0.38	-0.04±0.34
N10	-4.43 ±0.93	-1.3 ±0.49	-1.22 ±0.55	-0.03 ±0.23	0.11 ±0.28	0.03 ±0.3	-0.04±0.25	-0.04±0.41	-0.03±0.27
N5	-4.37 ±0.89	-1.05 ±0.44	-1.13 ±0.4	0 ±0.27	0.04 ±0.26	0.07 ±0.24	-0.01±0.23	0.06±0.27	0.03±0.29
**C**	-4.39 ±0.95	-1.08 ±0.29	-1.11 ±0.36	-0.04 ±0.25	0 ±0.24	0.05 ±0.26	-0.03±0.23	-0.01±0.27	-0.01±0.26
T5	-4.2 ± 0.98	-0.89 ±0.57	-1.19 ±0.5	0.02 ±0.26	0.07 ±0.22	-0.03 ±0.34	-0.06±0.28	-0.01±0.4	0.1±0.37
T10	-4.52 ±1.04	-1.01 ± 0.81	-1.21 ±0.7	0.07 ±0.31	0.01 ±0.35	0 ±0.32	0.02±0.32	-0.05±0.25	-0.01±0.29
T15	-4.33 ±1.34	-0.93 ±0.90	-1.13 ±0.86	0.03 ±0.34	0.11 ±0.31	-0.02 ±0.28	-0.07±0.27	0.06±0.38	-0.06±0.32
T20	-4.19 ±1.2	-0.99 ±1.04	-0.77 ±0.98	-0.07 ±0.38	-0.04 ±0.45	0.24 ±0.47	-0.04±0.36	-0.2±0.35	0.09±0.37
T25	-3.86 ±1.31	-0.79 ±1.05	-0.69 ± 1.02	-0.05 ±0.34	0.01 ±0.36	0.07 ±0.33	-0.01±0.36	-0.02±0.27	-0.01±0.3
T30	-3.63 ±1.35	-0.72 ±1.05	-0.5 ±1.13	-0.07 ±0.54	0.07 ±0.45	0.04 ±0.44	0.11±0.41	0.02±0.45	0.07±0.53
T35	-3.34 ±1.32	-0.57 ±1.14	-0.3 ±1.11	0.13 ±0.5	-0.25 ±0.58	0.19 ±0.4	-0.01±0.59	0.04±0.6	0.06±0.39

* Values are expressed in diopters (D). N is nasal visual field; T is temporal visual field; C is centre.

**Table 3.** Relative peripheral refractive error as mean spherical equivalent values (M±SD), horizontal astigmatism component ( *J*_0_ ± SD) and oblique astigmatism component ( *J*_45_ ± SD) for 27 eyes at different eccentricities under different conditions: without lens, Multistage PCL (Add +1.50D) and Proclear PCL (Add +1.50).

Eccentricity	*M*			*J*_0_			*J*_45_		
	WL ± SD Sig. (p)	Multistage PCL ± SD Sig. (p)	Proclear PCL ± SD Sig. (p)	WL ± SD Sig. (p)	Multistage PCL ± SD Sig. (p)	Proclear PCL ± SD Sig. (p)	WL ± SD Sig. (p)	Multistage PCL ± SD Sig. (p)	Proclear PCL ± SD Sig. (p)
N35	1.08±1.24 **< 0.001**	0.67 ± 1.23 **0.009**	1.13 ± 1.31 **< 0.001**	-0.33 ± 0.84 0.090	-0.51±1.05 **0.035**	-0.03±0.87 0.858	0.17±1.02 0.440	0.18±0.87 0.348	0.29±0.92 0.149
N30	0.8±1.1 **0.001**	0.35 ± 1.02 0.084	0.69 ± 1.16 **0.004**	0.4 ± 1.03 0.855	-0.05±0.82 0.747	0.18±0.69 0.226	-0.19±0.61 0.139	-0.16±0.72 0.288	0.17±0.87 0.355
N25	0.31±0.86 0.074	-0.05 ± 0.79 0.758	0.32 ± 0.91 0.075	0.1 ± 0.7 0.495	0.19±0.71 0.218	0.01±0.66 0.920	-0.19±0.7 0.190	-0.12±0.64 0.359	0.04±0.57 0.722
N20	0.09±0.57 0.429	-0.22 ± 0.5 0.032	-0.03 ± 0.66 0.808	0.05 ± 0.51 0.629	-0.07±0.55 0.551	-0.07±0.42 0.410	0.01±0.42 0.953	0.18±0.33 **0.014**	-0.14±0.38 0.084
N15	0.03 ± 0.43 0.717	-0.24 ± 0.35 0.001	-0.06 ± 0.5 0.568	0.02 ± 0.29 0.683	-0.05±0.38 0.544	-0.06±0.4 0.505	-0.03±0.3 0.674	-0.14±0.27 **0.018**	-0.03±0.38 0.746
N10	0.02 ± 0.35 0.716	-0.22 ± 0.46 0.025	-0.1 ± 0.41 0.209	0.03 ± 0.32 0.630	0.02±0.48 0.817	0.04±0.38 0.568	-0.04±0.23 0.386	-0.01±0.48 0.856	-0.02±0.33 0.692
N5	0.02 ± 0.19 0.578	0.03 ± 0.37 0.718	-0.02 ± 0.22 0.643	-0.01 ± 0.37 0.865	-0.05±0.33 0.491	0.04±0.31 0.487	-0.01±0.26 0.911	0.02±0.36 0.701	0.01±0.37 0.965
T5	-0.02 ± 0.31 0.708	0.19 ± 0.56 0.082	-0.08 ± 0.43 0.694	0.09 ± 0.28 0.125	0.01±0.35 0.927	-0.05±0.39 0.497	-0.06±0.37 0.428	-0.01±0.34 0.851	0.05±0.33 0.436
T10	-0.13 ± 0.50 0.196	0.07 ± 0.8 0.651	-0.1 ± 0.59 0.364	0.05 ± 0.32 0.421	-0.03±0.46 0.756	-0.03±0.46 0.741	0±0.35 0.972	-0.04±0.38 0.859	-0.03±0.39 0.729
T15	0.06 ± 0.92 0.733	0.15 ± 0.9 0.388	-0.02 ± 0.79 0.882	0.06 ± 0.33 0.398	0.07±0.42 0.444	-0.12±0.4 0.187	-0.08±0.28 0.144	0.11±0.46 0.236	-0.04±0.37 0.570
T20	0.2 ± 0.77 0.178	0.08 ± 1.04 0.707	0.34 ± 0.94 0.072	-0.02 ± 0.48 0.852	-0.11±0.43 0.247	0.11±0.6 0.414	-0.07±0.44 0.408	-0.18±0.36 **0.019**	0.16±0.38 0.054
T25	0.54 ± 0.90 **0.018**	0.29 ± 1.06 0.168	0.42 ± 0.95 **0.031**	-0.01 ± 0.37 0.894	-0.05±0.45 0.620	-0.01±0.53 0.948	-0.02±0.45 0.805	-0.03±0.42 0.730	-0.05±0.36 0.447
T30	0.76 ± 1.09 **0.001**	0.36 ± 1.06 0.089	0.64 ± 1.05 **0.004**	0.09 ± 0.42 0.310	0.01±0.38 0.819	0.01±0.58 0.918	0.1±0.42 0.250	-0.07±0.34 0.334	0.06±0.62 0.650
T35	1.06 ± 1.06 **< 0.001**	0.52 ± 1.14 **0.026**	0.81 ± 1.1 **0.001**	-0.01 ± 0.46 0.967	-0.36±0.58 **0.010**	0.08±0.36 0.301	-0.03±0.61 0.793	0.04±0.51 0.687	0.04±0.46 0.627

*Values are expressed in diopters (D). N is nasal visual field; T is temporal visual field; C is centre; p represents the value of statistical significance according to Paired t-test or Wilcoxon Signed Ranks Test. Bold indicates statistically significant power difference from central point (95% confid

A total of 27 myopic schoolchildren with a mean age of 14.18 ± 0.88 years (range: 13 years to 15 years) participated in this study. The mean central spherical equivalent refractive error was found to be -4.39 ± 0.95 D (range: -3.12D to -5.93D) without correction, with a mean axial length of 24.72 ± 0.92 mm (range: 23.51 mm to 26.39 mm). Table 2 presents the mean values of refractive error and standard deviations of eyes without contact lenses (WL), with Multistage PCL and Proclear PCL.

Table 3 shows the RPRE and standard deviations (SD) for mean spherical equivalent values ( *M*), horizontal astigmatism component ( *J*_0_) and oblique astigmatism component ( *J*_45_) in WL conditions, with Multistage PCL and Proclear PCL. A paired t-test showed that without contact lenses, there was a significant hyperopic defocus at and beyond 30° in the nasal visual field (N30° p= 0.001, N35° p< 0.001) and at and beyond 25° in the temporal visual field (T25° p= 0.018, T30° p= 0.001, and T35°< 0.001). When Multistage PCL was used, the peripheral defocus was only present at 35° in the nasal (p= 0.009) and temporal visual fields (p= 0.026). However, with Proclear PCL there was significant hyperopic defocus at and beyond 30° nasally (N30° p= 0.004, N35° p< 0.001) and at 25° temporally (T25° p= 0.031, T30° p= 0.004, and T35° p= 0.001). Multistage PCL shows a significant myopic defocus at nasal and temporal 35° for the *J*_0_ component, while for the *J*_45_ component, there was a significant hyperopic defocus at 20° but a significant myopic defocus at 15° nasal and 20° temporal.

With the Multistage PCL, the peripheral hyperopic defocus was decreased and only present at eccentricity of 35° nasally and temporally. However, with Proclear PCL, the hyperopic shift was still present at and beyond 30° in the nasal visual field and 25° in the temporal visual field. The hyperopic defocus was much smaller for Multistage PCL (+0.67 ±1.23 D at 35° nasal and +0.52 ±1.14 D at 35° temporal) as compared to Proclear PCL (+1.13 ±1.31 D at 35° nasal and +0.81 ±1.10 D at 35° temporal).

**Figure 1.**
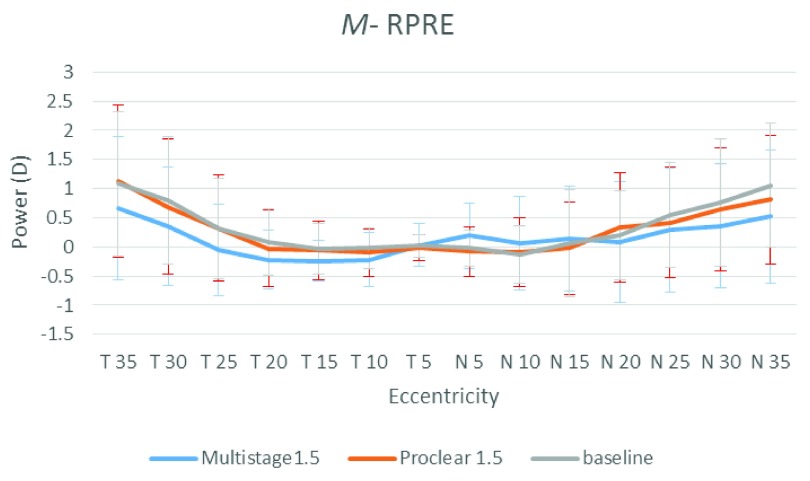
Baseline, Multistage PCL, and Proclear PCL of relative peripheral refractive error for mean spherical equivalent values ( *M*) at different eccentricities in the temporal (T) and nasal (N) visual fields.

**Figure 2.**
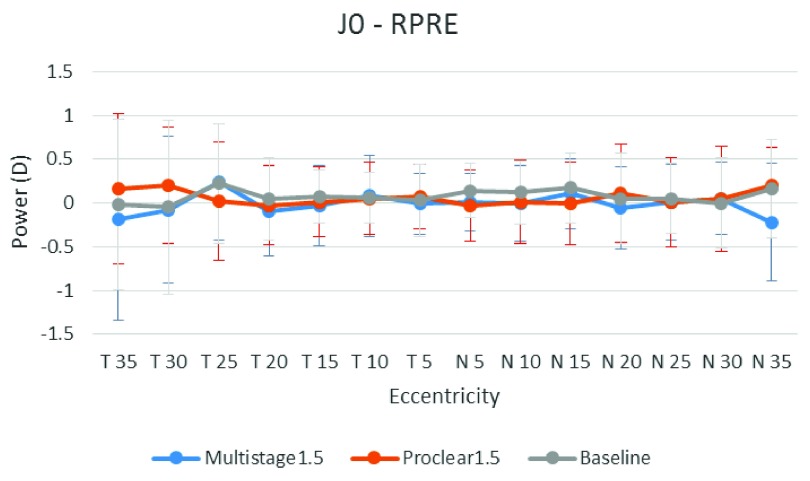
Baseline, Multistage PCL, and Proclear PCL of relative peripheral refractive error for horizontal astigmatism component values ( *J*_0_) at different eccentricities in the temporal (T) and nasal (N) visual fields.

**Figure 3.**
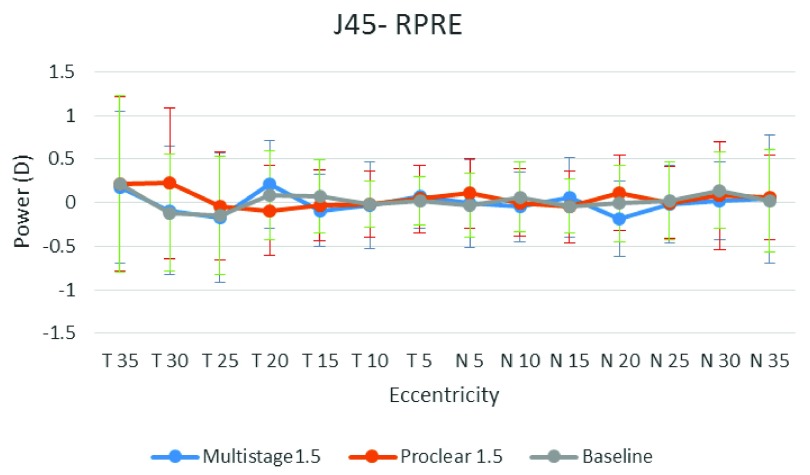
Baseline, Multistage PCL, and Proclear PCL of relative peripheral refractive error for oblique astigmatism component values ( *J*_45_) at different eccentricities in the temporal (T) and nasal (N) visual fields.

Figure 1, Figure 2 and Figure 3 illustrate the RPRE without contact lenses (baseline), with Multistage PCL and Proclear PCL for spherical equivalent value *M*, horizontal astigmatic component *J*_0_ and oblique astigmatic component *J*_45_, respectively. The hyperopic defocus is obvious at nasal and temporal visual fields with spherical equivalent value *M*. The *J*_0_ and *J*_45_, however, showed few changes at the peripheral field.

**Figure 4.**
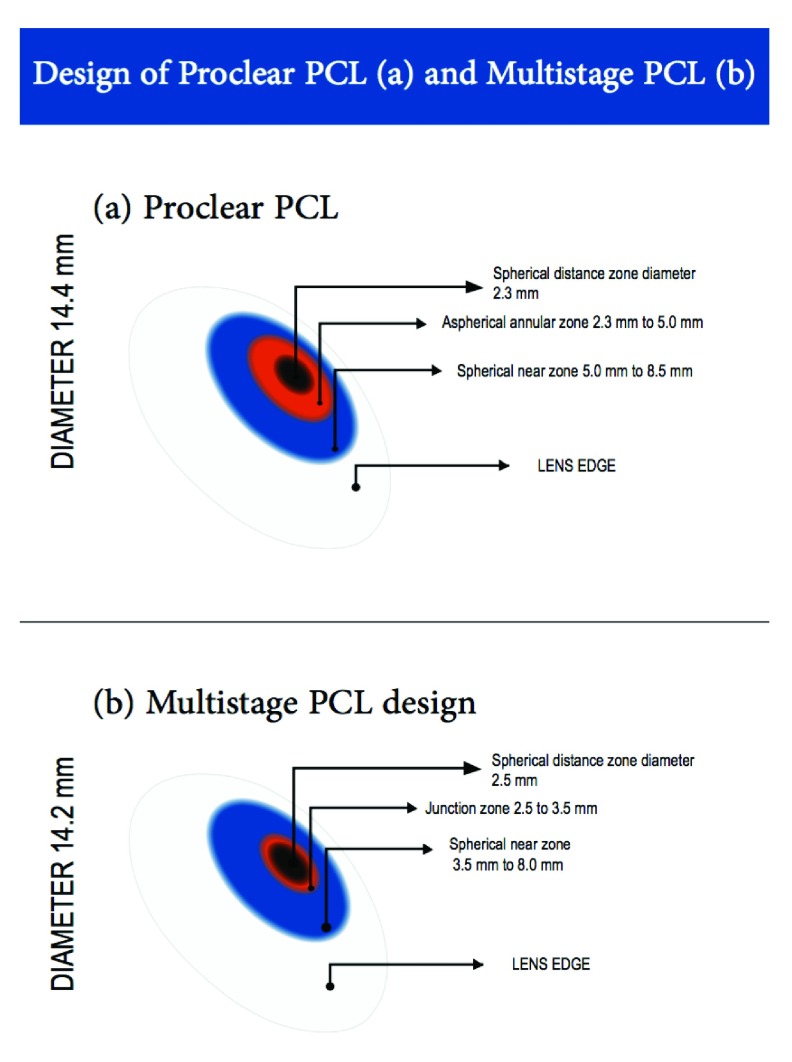
Progressive contact lens design ( **a**) Proclear progressive contact lens from Coopervision ( **b**) Multistage progressive contact lens from Seed.

A one-way repeated measures ANOVA was conducted to determine the changes in RPRE values for the mean spherical equivalent *M*, *J*_0_ and *J*_45_ components between the groups. The results of the ANOVA indicated a significant difference in mean spherical equivalent between groups with a Greenhouse–Geisser correction ( *F*(7.218, 43.794) = 4.285, p= 0.032). A post-hoc test using Bonferroni’s correction indicated a statistically significant difference in mean spherical equivalent RPRE ( *M*) between the baseline and Multistage PCL (p=0.015), while Proclear PCL showed no statistically significant different in comparison to the baseline (p=0.830). The results showed no statistically significant difference between without contact lenses and all contact lenses used in this study for *J*_0_ and *J*_45_ *F*(1.772, 52.926) = 0.871, p= 0.425, and *F*(0.440, 67.258) = 0.172, p=0.844, respectively. Therefore, it can be concluded that wearing a Multistage PCL can reduce hyperopic defocus in the retinal periphery.

## Discussion

With the extensive range of powers, materials and designs of soft contact lenses, they have become one of the most popular modes of myopia correction widely used by young adults. The present study compared the effect of RPRE along the horizontal visual field between two different designs of progressive contact lens (Multistage PCL and Proclear PCL). Although both progressive contact lenses in this study are simultaneous vision lenses, and had the same addition power (+1.50 D), the results showed a greater reduction in hyperopic defocus with Multistage PCL in comparison with Proclear PCL. The Multistage PCL had a decreased mean hyperopic defocus along the horizontal visual field up to 30° nasally and temporally, which indicated possible control of myopia progression for prolonged wear. However, the Proclear PCL showed significant hyperopic defocus from 30°, and 25° and beyond at the nasal and temporal visual fields, respectively.

The reason for the difference in hyperopic defocus at the periphery between both PCLs could be due to the difference in lens design. The Proclear PCL has a distance centre design, where the centre zone is 2.3 mm in diameter, and the added power increases progressively in a wide annular aspheric zone (from 2.3 to 5 mm/1.35 width), and ends in a spherical near zone (from 5 to 8.5 mm/1.75 width) where the full addition power of +1.50D exists. However, with the Multistage PCL, the design is different in diameters and power progression. The centre distance zone is 2.5 mm in diameter, surrounded by a narrow aspheric multifocal zone “junction zone” (from 2.5 to 3.5 mm/0.5 mm width), followed by a large spherical near zone (from 3.5 to 8.0 mm/2.25 width). With the dim illumination used in this study, the subject’s pupil size was approximately 4 to 5 mm. Hence, children were unable to view from the spherical near zone in Proclear PCL, where the near zone in this lens starts at 5 mm in diameter until 8 mm in diameter. However, with Multistage PCL the pupil size was sufficient to view the junction zone (2.5 mm to 3.5 mm), and part of the near spherical zone where the maximum addition power exists.

Phillips and Anstice ^15^ used dual-focus soft contact lenses on children aged 11 to 14 years old. The lens had a central distance correction zone followed by a concentric treatment zone with +2.00 D of peripheral retinal defocus. They reported a 36% reduction of myopia progression (-0.44 D versus -0.69 D) over 10 months of treatment as compared to a single vision contact lens. However, Sankaridurg *et al.* ^11^ 2011, found a reduction of myopia progression of 34% (-0.57 D versus -0.87 D) over one year of using multifocal contact lenses with a distance centre zone. The design had a progression increase of +2.00 D additional power compared to the control group. In 2013, Walline controlled myopia progression by 51% over 2 years of treatment by using Proclear PCL with +2.00 D additional power. However, the axial length elongation was slowed down by approximately 29% over this 2-year period ^16^. The authors could not explain why the myopia progression was slowed almost twice as much as the axial elongation. The reasons for not matching the myopia progression with the axial elongation in the Walline study might have been due to the fact that subjects were not randomly allocated to treatment groups, had a high drop-out rate (32.5%) with uncollected reasons for subjects’ withdrawals and there were 5 years of difference in data collection between the treatment group (June 2007 to May 2009) and the control group (September 2003 to Oct 2004).

Although neither PCL used in this study is made for myopia control, and they are commercially used for presbyopic older patients, the results of the present study illustrate no significant effect of relative peripheral hyperopic defocus with Proclear PCL +1.50D addition power. However, in 2013, Lopes-Ferreira *et al.* reported that a minimum addition of +2.00 D Proclear PCL D-design was necessary to induce a significant effect on peripheral refractive error, which explains why no statistical difference was found with Proclear PCL +1.50 D in the present study ^17^.

The mean central refractive error was -1.08 ±0.29D and -1.11 ±0.36D with Multistage PCL and Proclear PCL, respectively. Since the refractive error was fully corrected with contact lenses, the measurement of central refractive error was expected to be zero. This could be due to the infrared light beam used to measure the refractive error in the open-view Grand-Seiko WR-5100K autorefractometer. The size of the infrared light beam is about 2.3 mm in diameter, which is similar in size to the central zone of PCLs used in this study. Therefore, a small decentration of the lens (<0.5 mm) could have made the instrument read part of the addition power zone. However, by using the same procedure to measure all points of peripheral refraction with the same light beam, the relative peripheral refractive error would give the same myopic shift of readings, and therefore, the measurements were still valid and reliable along the 70° of the horizontal visual field.

## Conclusion

It was possible to decrease the peripheral retinal hyperopic defocus by using soft progressive contact lenses with a distance centre design. This study suggested that PCL designed with a narrow junction zone and wider spherical near zone had a greater effect on the pattern of peripheral refractive error, which may show better control of myopia in comparison to PCLs designed with a progressive increase of addition power.

## Data availability

The data referenced by this article are under copyright with the following copyright statement: Copyright: © 2016 Allinjawi K et al.

Data associated with the article are available under the terms of the Creative Commons Zero "No rights reserved" data waiver (CC0 1.0 Public domain dedication). 

*F1000Research*: Raw data for ‘Peripheral refraction with different designs of progressive soft contact lenses in myopes’, 2016, 10.5256/f1000research.9971.d143677 ^18^

## References

[1] Pan CW Ramamurthy D Saw SM: Worldwide prevalence and risk factors for myopia. *Ophthalmic Physiol Opt.* 2012;32(1):3–16. 10.1111/j.1475-1313.2011.00884.x 22150586

[2] Atchison DA Pritchard N Schmid KL: Peripheral refraction along the horizontal and vertical visual fields in myopia. *Vision Res.* 2006;46(8–9):1450–1458. 10.1016/j.visres.2005.10.023 16356528

[3] Mutti DO Hayes JR Mitchell GL: Refractive error, axial length, and relative peripheral refractive error before and after the onset of myopia. *Invest Ophthalmol Vis Sci.* 2007;48(6):2510–19. 10.1167/iovs.06-0562 17525178 PMC2657719

[4] Smith EL 3rd Kee CS Ramamirtham R: Peripheral vision can influence eye growth and refractive development in infant monkeys. *Invest Ophthalmol Vis Sci.* 2005;46(11):3965–3972. 10.1167/iovs.05-0445 16249469 PMC1762100

[5] Smith EL 3rd Ramamirtham R Qiao-Grider Y: Effects of foveal ablation on emmetropization and form-deprivation myopia. *Invest Ophthalmol Vis Sci.* 2007;48(9):3914–3922. 10.1167/iovs.06-1264 17724167 PMC2709928

[6] Smith EL 3rd Hung LF Huang J: Relative peripheral hyperopic defocus alters central refractive development in infant monkeys. *Vision Res.* 2009;49(19):2386–2392. 10.1016/j.visres.2009.07.011 19632261 PMC2745495

[7] Bakaraju RC Ehmann K Papas EB: Do peripheral refraction and aberration profiles vary with the type of myopia? - An illustration using a ray-tracing approach. *J Optom.* 2009;2(1):29–38. 10.3921/joptom.2009.29

[8] Tabernero J Vazquez D Seidemann A: Effects of myopic spectacle correction and radial refractive gradient spectacles on peripheral refraction. *Vision Res.* 2009;49(17):2176–2186. 10.1016/j.visres.2009.06.008 19527743

[9] Lin Z Martinez A Chen X: Peripheral defocus with single-vision spectacle lenses in myopic. *Optom Vis Sci.* 2010;87(1):4–9. 10.1097/OPX.0b013e3181c078f1 19826316

[10] Shankaridurg P Donovan L Varnas S: Spectacle lenses designed to reduce progression of myopia: 12-month results. *Optom Vis Sci.* 2010;87(9):631–641. 10.1097/OPX.0b013e3181ea19c7 20622703 PMC4696394

[11] Sankaridurg P Holden B Smith E 3rd: Decrease in rate of myopia progression with a contact lens designed to reduce relative peripheral hyperopia: one-year results. *Invest Ophthalmol Vis Sci.* 2011;52(13):9362–7. 10.1167/iovs.11-7260 22039230

[12] Lopes-Ferreira D Ribeiro C Maia R: Peripheral myopization using a dominant design multifocal contact lens. *J Optom.* 2011;4(1):14–21. 10.1016/S1888-4296(11)70035-8

[13] Rosén R Jaeken B Lindskoog Petterson A: Evaluating the peripheral optical effect of multifocal contact lenses. *Ophthalmic Physiol Opt.* 2012;32(6):527–534. 10.1111/j.1475-1313.2012.00937.x 22978717

[14] Thibos LN Wheeler W Horner D: Power vectors: an application of Fourier analysis to the description and statistical analysis of refractive error. *Optom Vis Sci.* 1997;74(6):367–375. 10.1097/00006324-199706000-00019 9255814

[15] Anstice NS Phillips JR: Effect of dual-focus soft contact lens wear on axial myopia progression in children. *Ophthalmology.* 2011;118(6):1152–1161. 10.1016/j.ophtha.2010.10.035 21276616

[16] Walline JJ Greiner KL McVey ME: Multifocal contact lens myopia control. *Optom Vis Sci.* 2013;90(11):1207–1214. 10.1097/OPX.0000000000000036 24061152

[17] Lopes-Ferreira D Ribeiro C Neves H: Peripheral refraction with dominant design multifocal contact lenses in young myopes. *J Optom.* 2013;6(2):85–94. 10.1016/j.optom.2013.01.001

[18] Allinjawi K Kaur S Akhir SM: Dataset 1 in: Peripheral refraction with different designs of progressive soft contact lenses in myopes. *F1000Research.* 2016 Data Source 10.12688/f1000research.9971.1 PMC5247792 28163898

