# Supplementary material for: Peripheral refraction with different designs of progressive soft contact lenses in myopes
Source: F1000Res. 2016 Nov 22;5:2742. [Version 1] doi: 10.12688/f1000research.9971.1 (PMC5247792; doi:10.12688/f1000research.9971.1)
Supplement: Peer review history

# Peer review: Referee response for version 1

**DOI:** 10.5256/f1000research.10746.r18870

**Published:** 2017-01-16

**Authors:** Ahmed Abdel-Kareem Mohamed Elmassry

**Affiliations:** 1 Department of Ophthalmology, Alexandria University, Alexandria, Egypt

*The title of the paper is appropriate for the contents of the article, as myopia is an error of refraction which is still a “hot topic “ and worth discussion.*

*The abstract of the paper is short and should include some more data in the Methods, results and Conclusions.*

*Measurement  of central and peripheral refraction by  using the open field auto-refractometer  needs short learning curve ,easy, non invasive fast method  for measuring refraction; these points should raise the sample size and age in the study.*

*The results are covered with good statistical analysis of data, reaching the conclusion of having the Multistage CL to be better than preclear CL.*

I have read this submission. I believe that I have an appropriate level of expertise to confirm that it is of an acceptable scientific standard.

# Peer review: Referee response for version 1

**DOI:** 10.5256/f1000research.10746.r18869

**Published:** 2017-01-13

**Authors:** Mustafa Abdu

**Affiliations:** 1 Department of Contact Lenses, Faculty of Optometry and Visual Science, Al-Neelain University, Khartoum, Sudan

Myopia is the most common type of refractive errors and it has become more prevalent recently. It has been reported that normal myopic eyes showing more hypermetropic (less myopic) values toward the peripheral retina.  In this paper, the authors attempt to find the effect of different new designs of progressive soft contact lenses in controlling peripheral refraction.

 Studying two different designs of lenses, the authors found that multistage PCLs had more effects on reducing the peripheral retinal hypermetropic defocus compared to Proclear PCLs which more likely could be due to the difference in designs between the two contact lenses.

 Power of the study:

 The power of this study could be due to:

1. Originality
2. Proper statistics and analysis

Technical comments:

1. Authors must highlight some additional findings in the result part of the abstract
2. Authors should write Lens specifications instead of lens design and materials in table (1)
3. Figure (4) should come earlier in the text body.

 

 Dr. Mustafa Abdu

 Assistant professor of Optometry

 Editor-in- Chief of Sudanese Journal of Ophthalmology (SJO)

I have read this submission. I believe that I have an appropriate level of expertise to confirm that it is of an acceptable scientific standard.
